# In for a penny, in for a pound: the effect of pre-engaging healthcare organizations on their subsequent participation in trials

**DOI:** 10.1186/s13104-015-1743-2

**Published:** 2015-12-08

**Authors:** Mirjam M. Garvelink, Adriana Freitas, Matthew Menear, Nathalie Brière, Dawn Stacey, France Légaré

**Affiliations:** CHUQ Research Centre, Hôpital St-Francois d’Assise, 10 Rue Espinay, Quebec City, QC G1L 3L5 Canada; Centre de santé et de services sociaux de la Vieille-Capitale, Quebec City, QC Canada; Ottawa Hospital Research Institute and Faculty of Health Sciences, University of Ottawa, Ottawa, Canada; Department of Family Medicine and Emergency Medicine, Université Laval, Quebec City, QC Canada

**Keywords:** Integrative knowledge translation, Study recruitment, Clinical trials, Shared decision making, Caregivers and the frail elderly

## Abstract

**Background:**

Participant recruitment in clinical trials is often challenging. Building partnerships with healthcare organizations during proposal development facilitates access to the community and may influence its subsequent organization participation and participant recruitment. We aimed to assess how pre-engaging directors of homecare organizations influenced organization participation in a subsequent trial.

**Findings:**

Repeated cross-sectional study prior to a cluster randomized controlled trial involving 33 eligible Health and Social Services Centres (HSSCs). During proposal development, we asked eligible HSSC directors in a randomized order about their willingness to participate in our trial, if funded. In the pre-engagement phase, 23 directors were contacted until we met sample size requirements (n ≥ 16); 19 of whom wrote letters of intent. Once funded, we contacted all 33 eligible HSSC directors in a randomized order to enroll them. Of the 19 directors who provided letters of intent, 15 agreed to participate (79 %); of the four who did not provide letters, one agreed to participate (25 %); and of the ten who had not been approached in the pre-engagement phase, two agreed to participate (20 %). Fisher exact tests indicated that providing letters of intent was associated with subsequent participation (p = 0.003).

**Conclusions:**

Given that significantly more HSSCs directors who signed letters of intent followed through with study participation, pre-engagement with trial sites during proposal development appears to improve recruitment.

## Findings

### Background

Recruitment of patients [1] and healthcare providers [2] for clinical trials is often challenging. The two problems seem to be interrelated, as low recruitment rates are more often due to healthcare providers’ reluctance to enter patients in trials than to patients’ reluctance to participate [3]. A systematic review on factors that influence clinicians’ willingness to invite patients to join a study indicated an association between clinicians’ motivation to participate and subsequent patient recruitment. Clinicians’ sense that they were active members of a research group positively affected their recruitment of patients [4]. Recruitment has also been favourable if healthcare professionals (a) have positive attitudes about the intervention and study design, (b) are interested in the research topic [4], and (c) receive clear communication or training on the methods used in the trial [5]. In this era of patient and public engagement in healthcare, greater engagement of patients and professionals in research is attracting widespread interest [6] and for numerous grant competitions, partnerships with stakeholders are now required [7].

During the development of research proposals, pre-engagement with healthcare providers (one of three key elements of integrative knowledge translation [7]) is also thought to positively influence their own subsequent participation (active involvement) and retention in the study, as reciprocal relationships have already been initiated [8]. Their participation can be especially meaningful in research projects that intend to change the current course of practice [8], as they are the ones targeted to adopt the change. Several phases can be distinguished in building partnerships with healthcare providers: pre-engagement; engagement; assessment, reflection and feedback; and ongoing maintenance [8]. However, little is known about the influence of each phase on study-related outcomes (e.g. on healthcare organizations’ intention to participate, participation rates of healthcare teams) [8].

We hypothesized that by focusing on the pre-engagement of healthcare organizations, we could address provider-related recruitment problems which could in turn influence their patient recruitment later on. This study was embedded in a multicenter cluster randomized controlled trial (RCT) aiming to implement an interprofessional approach to shared decision making (IP-SDM) in homecare teams regarding decisions about location of care among the frail elderly [9]. In the context of developing a research proposal for this trial, we evaluated the effect of pre-engaging directors at eligible organizations providing homecare services on the subsequent participation (engagement) of their homecare teams in our funded research project.

## Methods

This repeated cross-sectional study was the preliminary phase of a multicenter cluster RCT conducted with Health and Social Services Centres (HSSCs, the main public sector providers of homecare) and their homecare teams in the Province of Quebec. A detailed protocol was published elsewhere [9]. The aim of the present study was to analyze the association between pre-engagement of HSSCs and their subsequent participation in the cluster RCT.

The study was conducted in a network of HSSCs served by Laval University, including 45 rural and urban HSSC in Central and Eastern regions of the province of Quebec, Canada. In order to guarantee feasibility with regard to our budget and recruitment of a sufficient number of frail elderly clients eligible for the cluster RCT (i.e. facing the decision about location of care), eligibility criteria for HSSCs were that: (1) they served a geographical area with a population of over 10,000 inhabitants; and that (2) their distance from Quebec City (location of research team) was less than 500 km.

This study was approved by the ethics committee of Centre Hospitalier Université de Quebec Research Centre (CRCHUQ), and all HSSCs that accepted to participate in the trial. For enrolled and thus participating HSSCs, only health providers and clients are asked to complete informed consent.

### Procedures

#### Pre-engagement

During study proposal development, a computer generated random list of the order in which to contact directors of eligible HSSCs was created. In contacting directors, we emailed a draft abstract of the study proposal and scheduled a telephone call with a team member (AF) to explain the study and answer their questions. HSSC directors were informed that participation would involve their organization being randomized between usual care and receiving a multifaceted IP-SDM intervention consisting of training in IP-SDM, a tutorial and a decision aid to use in practice [9]. In addition, all participating HSSCs were asked to designate a person to liaise between the homecare and research teams.

Based on sample size calculations for our main outcome of interest (i.e. involvement of the frail elderly in decisions about location of care), we estimated that 16 HSSCs that agreed to participate would allow us to recruit the desired number of patients in the subsequent RCT. In anticipation of possible drop-outs, we initially contacted more directors in order to get at least 16 HSSCs to agree to participate. Hence, to ensure meeting our sample size requirement, 23 of the 33 eligible HSSCs were contacted during the pre-engagement phase, to ask for a letter describing their support for the study and their intention to participate should it be funded.

#### Engagement

Once funding was approved, we contacted all directors of eligible HSSCs (n = 33) in a randomized manner to invite their homecare teams to participate in the study. Directors were contacted using the same process as above, only now they had contact with the principal investigator (PI), and they were informed that the study was funded. They were then asked to indicate whether they (still) agreed to participate.

### Statistical analysis

We conducted Fisher exact tests to assess whether providing a letter of intent during proposal development was associated with subsequent participation. Results were considered significant if p ≤ 0.01.

## Results

### HSSCs approached during proposal development (pre-engagement)

**Fig. 1:**
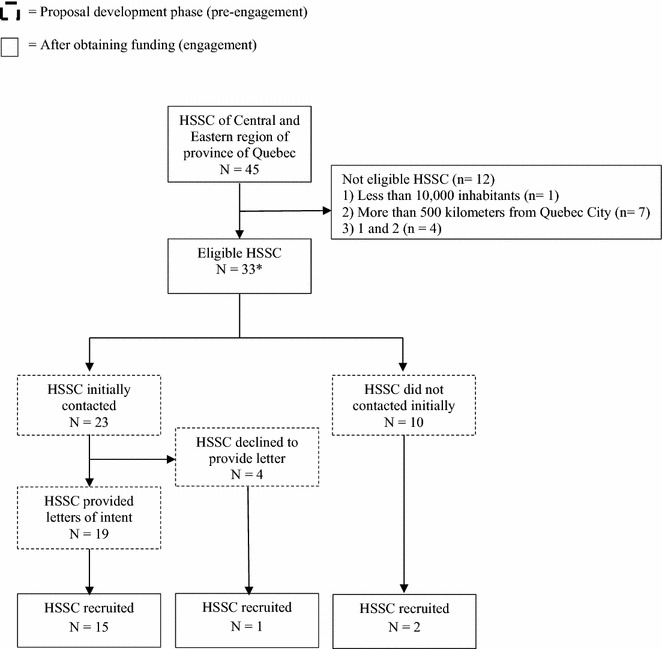
Recruitment flow chart. * All 33 eligible HSSCs were contacted once the project was funded

In total, among 45 HSSCs in Central and Eastern regions of the Province of Quebec (i.e. the network served by Laval University), 33 HSSCs were eligible. During the proposal development phase, we contacted 23 HSSC directors, 19 of whom wrote letters of intent (83 %) (Fig. 1). Reasons HSSC directors did not provide a letter of intent were internal restructuring (n = 3), and their involvement in other research projects (n = 1).

### HSSCs approached after funding received (engagement)

After receiving funding for the study, we contacted the directors of all 33 eligible HSSCs in a randomized order (Table 1), including those who had signed a letter of intent (n = 19), those who had declined to sign one (n = 4), and those who had not yet been approached (n = 10). In total, 18 HSSC directors agreed to participate in the study, including 15 who had provided letters of intent, one who initially refused to provide a letter of intent, and two others newly approached (Fig. 1). Overall, significantly more homecare teams in HSSCs whose directors provided letters of intent agreed to participate in the study (p = 0.003) than in those whose directors had not provided letters of intent, or were not originally approached (Table 1).

**Table 1:** HSSCs participation rates

	Initial letter of intent N (%)	Participated after funds assured N (%)
Provided letter	19 (83)	15 (79)*
Declined to provide letter	4 (17)	1 (25)
Not approached at baseline	10 (n/a)	2 (20)
Total	33	18 (55)

* p < .01

In our contacts with HSSC directors many expressed their interest in the research topic and mentioned that it was very much in line with the current focus of their HSSC (i.e. patient-centered care, interprofessional approaches, senior support). In addition, homecare teams showed a marked interest in receiving the IP-SDM training (part of the intervention arm), and even asked about the possibility of receiving the training (once the study was completed) even if they were randomized to the control group.

## Discussion

Our experience with pre-engaging HSSCs (by informing directors of the trial and asking them to provide letters of intent) indicated that this seemed to affect the homecare team’s subsequent participation in our research project. We observed that participation rates were significantly higher in HSSCs that provided us with a letter of intent during the pre-engagement phase than in HSSCs that did not. This suggests that in designing and implementing clinical trials requiring enrollment of patients and their providers, it is worthwhile to pre-engage with directors of the healthcare organizations. Moreover, the required time, resources and efforts to obtain letters of intent from HSSC directors was well invested. In fact, obtaining them not only strengthened the grant proposal by demonstrating feasibility to peer review committees but also indicated to funders the feasibility of the intended collaboration, an increasingly important requirement for funding opportunities [7, 10]. Moreover, it seemed to increase homecare teams’ willingness to participate. Receiving requests for letters of intent, for the healthcare organizations, is a first opportunity for them to connect with researchers, and gives them time to learn about a project, make a commitment to it, and plan ahead for the practical details of their involvement. However, anticipation on loss to follow-up is yet important, as, at the moment of writing this manuscript, two HSSCs of whom the direction originally agreed to participate and who wrote letters of intent refrained from participating because the healthcare team involved did not want to add an extra study to their workload. So anticipating loss of follow-up by approaching more HSSC than originally needed turned out to be a good strategy.

Two key limitations should be considered. Although our results show a significant association between directors signing a letter of intent and their subsequent participation, we cannot rule out the influence of other factors such as the subject of research, credibility of the research team, and the procedure of involving the teams. It should be noted that there were no previous ties between the research team and the HSSCs that were contacted except for the ties with the HSSC in which the PI works. However, the fact that she works for an HSSC and hence can be seen as a peer or colleague, may have positively influenced the commitment of other HSSCs with the research project. It would be interesting to measure the effect of a letter of intent on more aspects of study participation and to explore the influence of related factors. Second, our sample size was small, which prohibited a more thorough statistical analysis. However, even with our small sample size, we found a highly significant *p* value.

In conclusion, our experience suggests the importance of building partnerships and securing commitments from healthcare organizations during the pre-engagement phase of projects to improve subsequent study participation. Future research should assess which strategies are the most effective for initiating successful partnerships with health organizations before securing funding for research and should evaluate the exact impact of such partnerships on the subsequent phases of research initiation, such as recruitment, follow-up, and ongoing maintenance.

## Availability of supporting data

The data set supporting the results of this article is included within the article (Table 1; Fig. 1).

## Abbreviations

IP-SDM: interprofessional approach to shared decision making
HSSCs: Health and Social Services Centres
PI: principal investigator
RCT: randomized controlled trial

## References

[1] Treweek S, Pitkethly M, Cook J, Kjeldstrom M, Taskila T, Johansen M (2010). Strategies to improve recruitment to randomised controlled trials. Cochrane Database Syst Rev.

[2] Asch S, Connor SE, Hamilton EG, Fox SA (2000). Problems in recruiting community-based physicians for health services research. J Gen Intern Med.

[3] Fallowfield L, Ratcliffe D, Souhami R (1997). Clinicians’ attitudes to clinical trials of cancer therapy. Eur J Cancer (Oxford, England: 1990).

[4] Rendell JM, Merritt RD, Geddes JR (2007). Incentives and disincentives to participation by clinicians in randomised controlled trials. Cochrane Database Syst Rev.

[5] Fletcher B, Gheorghe A, Moore D, Wilson S, Damery S (2012). Improving the recruitment activity of clinicians in randomised controlled trials: a systematic review. BMJ Open..

[6] Fleurence RL, Forsythe LP, Lauer M, Rotter J, Ioannidis JPA, Beal A (2014). Engaging patients and stakeholders in research proposal review: The Patient-Centered Outcomes Research Institute engaging patients and stakeholders in research proposal review. Ann Intern Med.

[7] Canadian Institutes of Health Research. Guide to knowledge translation planning at CIHR: integrated and end-of-grant approaches. Ottawa, Ontario: Canadian Institutes of Health Research. 2012. Contract No.: ISBN 978-1-100-20517-5.

[8] Ochocka J, Moorlag E, Janzen R (2010). A framework for entry: PAR values and engagement strategies in community research.

[9] Légaré F, Brière N, Stacey D, Bourassa H, Desroches S, Dumont S, Fraser K, Freitas A, Rivest L, Roy L (2015). Improving the decision making process about location of care with the frail elderly and their caregivers: study protocol for a cluster randomized trial (the DOLCE study). Trials..

[10] Bliss DZ (2010). Letters of support for a research grant proposal. J Wound Ostomy Cont Nurs Off Publ Wound Ostomy Cont Nurses Soc WOCN..

